# Intramitochondrial Src kinase links mitochondrial dysfunctions and aggressiveness of breast cancer cells

**DOI:** 10.1038/s41419-019-2134-8

**Published:** 2019-12-09

**Authors:** Marie-Ange Djeungoue-Petga, Olivier Lurette, Stéphanie Jean, Geneviève Hamel-Côté, Rebeca Martín-Jiménez, Marine Bou, Astrid Cannich, Patrick Roy, Etienne Hebert-Chatelain

**Affiliations:** 1 Canada Research Chair in Mitochondrial Signaling and Physiopathology, Moncton, NB Canada; 2 Department of Biology, Université de Moncton, Moncton, NB Canada; 3 Endocannabinoids and NeuroAdaptation, NeuroCentre, INSERM U1215 Bordeaux, France; 4 Université de Bordeaux, Bordeaux, France; 5 Department of Chemistry and Biochemistry, Université de Moncton, Moncton, NB Canada

**Keywords:** Mechanisms of disease, Phosphorylation

## Abstract

High levels and activity of Src kinase are common among breast cancer subtypes, and several inhibitors of the kinase are currently tested in clinical trials. Alterations in mitochondrial activity is also observed among the different types of breast cancer. Src kinase is localized in several subcellular compartments, including mitochondria where it targets several proteins to modulate the activity of the organelle. Although the subcellular localization of other oncogenes modulates the potency of known treatments, nothing is known about the specific role of intra-mitochondrial Src (mtSrc) in breast cancer. The aim of this work was to determine whether mtSrc kinase has specific impact on breast cancer cells. We first observed that activity of mtSrc is higher in breast cancer cells of the triple negative subtype. Over-expression of Src specifically targeted to mitochondria reduced mtDNA levels, mitochondrial membrane potential and cellular respiration. These alterations of mitochondrial functions led to lower cellular viability, shorter cell cycle and increased invasive capacity. Proteomic analyses revealed that mtSrc targets the mitochondrial single-stranded DNA-binding protein, a regulator of mtDNA replication. Our findings suggest that mtSrc promotes aggressiveness of breast cancer cells via phosphorylation of mitochondrial single-stranded DNA-binding protein leading to reduced mtDNA levels and mitochondrial activity. This study highlights the importance of considering the subcellular localization of Src kinase in the development of potent therapy for breast cancer.

## Introduction

Breast cancer (BC) is characterized by various phenotype, clinical outcome, and response to treatment. BC can be classified into different subtypes based on the expression of the estrogen receptor (ER), progesterone receptor (PR) and the epidermal growth factor receptor 2 (HER2)^1^. For instance, ER and/or PR receptor-positive tumors are treated with endocrine therapy whereas aggressive HER2-positive diseases are candidates for trastuzumab, a monoclonal antibody against HER2^2,3^. In 15% of breast tumors, none of these receptors are amplified. These so-called triple negative (TN) breast cancer are thus not responsive to trastuzumab or endocrine therapy and have a poor prognosis^2,3^. Several attempts were made to better characterize the molecular processes linked to heterogenous BC and generate potent and specific treatment to non-responding BC.

Considering the role of metabolic pathways in cancer pathophysiology, numerous mitochondria-targeted strategies were developed for the treatment of cancer, including BC. Several cancer cells exhibit lower oxygen consumption and higher glycolysis activity^4,5^ and mitochondria are crucial components in this metabolic signature. These organelles play key functions in cell physiology, including generation of ATP, reactive oxygen species (ROS), apoptosis and calcium homeostasis. Their functions are however readjusted in several types of cancer to sustain unlimited cell growth and proliferation, inhibition of apoptosis and intense anabolism^5^. Mitochondria appears metabolically dysfunctional in several cancer types, such as BC^6,7^. OXPHOS and ATP levels are decreased in breast cancer cells as compared to normal breast epithelial cells^6^. Altered oxidative phosphorylation (OXPHOS) can lead to upregulation of ROS levels and contribute to malignant transformation via the accumulation of oncogenic DNA mutations and activation of oncogenic signaling pathways^8^. The rewiring of mitochondrial metabolism also support high proliferation rates of various cancer cells. Several tricarboxylic (TCA) cycle intermediates, including alpha-ketoglutarate and citrate, are exported out of mitochondria to be used for biosynthesis of lipids, amino acids and nucleotides in cancer cells^9^. The metabolic phenotype is however variable among subtypes of BC cells. Triple negative BC (TNBC) cells have lower mitochondrial activity and higher glycolysis than other subtypes^7^. TNBC cells MDA-MB-231 and MDA-MB-436 have a more fragmented mitochondrial reticulum, leading to increased migration and invasion capacities than the ER- and PR-positive MCF7 cells^10^. The glutaminase inhibitor CB-839 decreases proliferation of HCC-1806 and MDA-MB-231 TNBC cells without affecting ER-positive T47D cells^11^. These findings suggest that differential metabolic signatures of BC subtypes should be considered as potential therapeutic targets.

The c-Src kinase (named hereafter Src) oncogene is a well-known therapeutic target for various type of cancer including BC. Src is a central messenger in several important signaling pathways involved in proliferation, differentiation, survival, motility, and angiogenesis^12,13^. High expression and/or activity of Src is observed in several types of solid tumors, including BC tissue, where it is associated to increased invasiveness and metastatic potential, and with lower survival of patients^14–16^. Conversely, genetic or pharmacological disruption of Src activity decreases survival, migration, invasion and proliferation in BC cells^17–21^. Hence, several inhibitors of Src were developed and tested for the treatment of cancer and tumors^22–24^. The impact of Src dysregulation appears however different among subtypes of breast cancer cells. For instance, the Src inhibitor dasatinib decreases the growth of TNBC cells without clear effect on other subtypes^19,20^. This different responsiveness of breast cancer sub-groups to therapies requires a better comprehension of the molecular machinery associated with the tumorigenic phenotype of breast cancer cells. Src is localized in multiple subcellular compartments, including nucleus, plasma membrane and mitochondria^12,25–27^. Interestingly, intra-mitochondrial Src (mtSrc) is present in different types of cancer cell, including BC cells^26,27^. The mtSrc targets various proteins involved in different mitochondrial functions, ranging from generation of ATP, production of ROS to apoptosis^25,26,28–30^. Although Src and mitochondria are both important in the pathophysiology of BC, the role of mtSrc among BC subtypes remains scantly studied.

The aim of this work was to describe the role of mtSrc in metabolic and neoplasic phenotype of BC cells. To address this, we first characterized (mt)Src levels and activity in 12 control and BC cell lines. Our results show that mtSrc activity is highest in TNBC cells. To determine the precise role of mtSrc, we expressed a mitochondria-targeted mutant of Src in several BC cells. Our findings demonstrate that over-expression of Src only in mitochondria is sufficient to alter mitochondrial metabolism, viability, proliferation and invasive capacities. This study also demonstrates that mtSrc induces mitochondrial dysfunctions and aggressiveness via phosphorylation of mitochondrial single-stranded DNA-binding protein and reduced mtDNA levels. Globally, this work provides a mechanistic explanation of why TNBC cells are more sensitive to Src inhibitors and suggests that targeting mtSrc signaling should be considered in the treatment of BC.

## Methods

### Material

BT-483, SK-BR-3, MDA-MB-453, BT-549, hTERT-HME-1, MDA-MB-468, and HCC-1954 cell lines were obtained from American Type Culture Collection (VA, USA). MDA-MB-231, MCF-7, T-47D, MCF-10A and MCF-12A cell lines were generously provided by the Atlantic Cancer Research Institute (Moncton, NB, Canada). Epidermal growth factor (EGF), bovine serum albumin (BSA) and actinomycin D (ACT001.5) were obtained from Bioshop (ON, Canada). Mouse monoclonal antibodies specific for HA-Tag (2367), phospho-tyrosine (94115), α-tubulin (3873), and rabbit monoclonal antibodies specific for Src (2123), phospho-Src Family Tyr-419 (6943), EGFR (4267), SOD2 (13141) were purchased from Cell signaling (USA). Mouse monoclonal antibodies specific for COXII (156031), UQCRC2 (14745), NDUFA9 (14713), ATP5A (110273), cytochrome c (110325) and SDHa (14715) were obtained from Abcam (Cambridge, UK). Mouse monoclonal antibody Src-B12 (8056) and rabbit polyclonal specific for TOM20 (11415) were purchased from Santa Cruz Biotechnology (Dallas, TX, USA). The horseradish peroxidase-conjugated F(ab′)_2_ fragment of anti-mouse (115-035-003) and anti-rabbit (111-035-003) were purchased from Jackson ImmunoResearch Laboratories (West Grove, PA, USA). Oligomycin (O4876), rotenone (R8875), antimycine A (A8674) and carbonylcyanide-*p*-fluoromethoxyphenylhydrazone (FCCP, C2920) were purchased from Sigma (MO, USA). Annexin V-FITC (640906) was purchased from Biolegend (CA, USA) and propidium iodide (PI, 40017) from Biotium Inc. (CA, USA). KI-67-FITC was purchased from Thermo Fisher Scientific (MA, USA).

### Cell culture

MCF-10A and MCF-12A cells were maintained in culture with high glucose (4.5 g/L) Dulbecco’s modified Eagle’s medium (DMEM) and Ham’s F12 medium, respectively, supplemented with 20 ng/mL human epidermal growth factor, 100 ng/mL cholera toxin, 10 µg/mL bovine insulin, 500 ng/mL hydrocortisone, 500 ng/mL hydrocortisone, 2 mM glutamine, 1 mM pyruvate, 10% (v/v) FBS, 100 units/mL penicillin and 100 µg/mL streptomycin. BT-483 cells were maintained in RPMI-1640 supplemented with 2 mM glutamine, 1 mM pyruvate, 10% (v/v) of FBS and penicillin-streptomycin. Other cell lines were maintained in culture with high glucose (4.5 g/L) DMEM supplemented with 2 mM glutamine, 1 mM pyruvate, 10% (v/v) of FBS and penicillin-streptomycin. MDA-MB-231 and BT-549 cells were also maintained in culture with DMEM without glucose supplemented with 2 mM glutamine, 10 mM galactose, 1 mM pyruvate, 10% (v/v) of FBS and penicillin-streptomycin. All cell lines were kept at 37 °C in 5% CO_2_ and 95% humidity. Cells were maintained in their respective medium for at least five passages before analysis and the medium was renewed every 2 to 3 days. Analysis and cell harvesting were always performed when confluency was about 80–90%.

### Plasmids

To generate Src-HA and MLS-Src-HA constructs, the sequence of Src was amplified from pCMV5-Src (Addgene 13663) and fused to the sequence of human influenza hemagglutin (HA) and the mitochondrial leading sequence of cytochrome c oxidase subunit VIIIa. The Src-GFP mutant was kindly provided by Yoav Henis^31^ (Tel Aviv University, Israel). The phospho-deficient mutant mtSBB-Y73F was generated by PCR amplification of pCMV6-Entry-mtSBP from OriGene (MD, USA) using 5′-GGGGATAGTGAAGTTTTCCAACTGGG-3′ and 5′-CTCCAGCTTGGTTCCCAATAGACC-3′ primers. Plasmids were transfected using polyethylenimine (PolySciences, PA, USA). Cells were analyzed 48 h following transfection.

### Isolation of mitochondria

Mitochondrial fractions were isolated as described previously^32^. Briefly, cells were harvested, resuspended in isolation buffer (250 mM sucrose, 1 mM EDTA, 5 mM HEPES, pH 7.4) containing protease and phosphatase inhibitor cocktails and disrupted with 15 strokes using a 25-gauge syringe. The cell debris and nuclei were removed by centrifugation at 1,500 g for 5 min (4 °C). The supernatant was considered as total cell lysate (TCL) and was centrifuged at 12,500 g for 10 min (4 °C). Then, the obtained supernatant was kept and considered as the cytosolic fraction (Cyto) whereas the pellet was resuspended. The centrifugation cycle was repeated and mitochondria-enriched fractions (Mito) were obtained from the last pellet.

### Gel electrophoresis and Western Blot

Samples were diluted in SDS-PAGE sample buffer (62.5 mM Tris 1 M, pH 6.8; 10% (v/v) glycerol, 2% (w/v) sodium dodecylsulfate (SDS), 0.5% Bromophenol blue, 2.5% (v/v) β-mercapto-ethanol) and boiled at 95 °C during 5 min. Samples were then separated using 7, 10 or 12% SDS-polyacrylamide mini-gel at 300 V during 30 min. Proteins were blotted to polyvinylidine difluoride (PVDF) membranes. Membranes were blocked for 1 h in TBST containing 5% BSA, and incubated with various primary antibodies. Lastly, membranes were incubated for 1 h with horseradish peroxidase-conjugated F(ab′)_2_ fragment of anti-mouse or anti-rabbit. Immunoblots were visualized by chemiluminescence using the ChemiDoc Touch imaging system (Biorad, CA, USA). Immunolabelings were quantified by densitometric analysis using ImageJ (NIH, MD, USA).

### Confocal microscopy

Cells seeded on 18-mm round glass coverslips transfected and treated as indicated were placed on the stage of the Olympus FV1000 confocal fluorescence microscope (Tokyo, Japan) and imaged using a 60X oil objective (UPLAN 60x oil, 1.35NA, Olympus), and appropriate excitation laser and filters. For each experiment, 50 cells were randomly selected and analyzed. Stacks of 30 images separated by 0.2 μm along the z axis were acquired. Three-dimensional reconstruction and volume rendering of the stacks were carried out with the appropriate plug-in of ImageJ (NIH).

### Oxygraphic measurements

Oxygen consumption assays were performed using the high-resolution respirometry system Oxygraph-2k Oroboros (Innsbruck, Austria). Cell respiration was measured with 4 × 10^5^ cells ml^−1^ according to volume-specific flux at 37 °C in 2 mL chambers at a stirring rate of 750 rpm. Three different states of endogenous respiration with intact cells were measured: (i) basal respiration representing the endogenous physiological coupled state, (ii) respiration with oligomycin (2 μg mL^−1^) representing the non-coupled resting respiration, and (iii) maximal uncoupled respiration induced by FCCP (0.5 μM steps with 2.5 μM final concentration) providing a measure of the maximal capacity of ETS under conditions of physiological substrate supply in the intact cells.

### Mitochondrial membrane potential, ROS production and mitochondrial mass

Mitochondrial membrane potential was examined using tetramethylrhodamin, methyl ester (TMRM, LifeTechnologies, CA, USA). Briefly, cells were rinsed with PBS and incubated with 10 nM TMRM during 15 min at 37 °C in 5% CO_2_ and 95% humidity. For all experiments, we confirmed that TMRM fluorescence was completely abolished following co-incubation with the mitochondrial uncoupler FCCP (1.5 µM) (data not shown).

Oxidative stress was evaluated using MitoSox™ (Life Technologies, CA, USA). Briefly, cells expressing the different constructs were pre-incubated with vehicle or 0.5 µM rotenone and 2.5 µM antimycin A during 30 min at 37 °C in 5% CO_2_ and 95% humidity. Upon treatment, cells were rinsed by PBS and incubated with 5 µM MitoSox™ during 45 min at 37 °C in 5% CO_2_ and 95% humidity.

Mitochondrial mass was evaluated using Mitotracker Green™ (Life Technologies, CA, USA). Briefly, cells were incubated with 150 nM Mitotracker Green™ during 30 min at 37 °C in 5% CO_2_ and 95% humidity.

After incubation with probes, cells were rinsed and imaging was performed. TMRM, MitoSox™ and Mitotracker Green™ fluorescence were examined using the EVOS FL Auto 2 imaging system (Thermo Fisher Scientific, MA, USA) with a ×40 objective (LPLAN 40 × , 0.65NA, EVOS). For each independent experiment, total fluorescence intensity was quantified for 50 cells using ImageJ (NIH, MD, USA).

### ATP production

The intracellular ATP production assay was assessed using the ATP bioluminescence assay kit HS II from Sigma (MO, USA) as described previously^33^. Briefly, 1 × 10^6^ of cells were harvested in 1 mL DMEM medium. Cells were treated with vehicle or rotenone (5 µM) and antimycin A (2.5 µM) during 1 h at 37 °C under stirring. Then, cells were lysed using a boiling lysis buffer provided with the kit during 5 min. Light emitted was detected and quantified using the Varioskan plate reader (Flash version 2.4.5, Thermo Fisher, MA, USA). The ATP content derived from mitochondria was determined by subtracting ATP levels obtained in cells treated with rotenone and antimycin A from the ATP levels obtained in untreated cells (ATPmito = ATPtotal − ATProtenone + antimycin A).

### Immunoprecipitation

The phosphorylation status of the cytochrome c oxidase subunit II (COXII) was examined using immunoprecipitation. Briefly, cells were harvested and resuspended in a non-denaturing lysis buffer (20 mM Tris HCl pH 8, 137 mM NaCl, 10% glycerol, 1% Triton X-100, 2 mM EDTA) in the presence of 1% protease inhibitor cocktail (PIC002) (Bioshop, ON, Canada) and 2 mM sodium orthovanadate. Following lysis, samples were centrifuged at 12,500 *g* during 10 min (4 °C) to remove debris. Immunoprecipitation was performed on 2 mg of protein with the antibody COXII overnight at 4 °C. Protein A/G agarose beads (20 µL, Santa Cruz, sc-2003) were then added and incubation continued during 4 h at 4 °C under continuous agitation. Beads were washed three times with non-denaturing lysis buffer and elution was performed with SDS-PAGE sample buffer during 5 min at 95 °C. Samples were then processed for western blotting.

### Apoptosis assays

Apoptosis was measured in cells labeled with Annexin V-FITC and PI using flow cytometry. Briefly, cells were incubated with vehicle or actinomycin D (5 µM) during 48 h. Cells were then harvested and resuspended in Annexin V binding buffer (Biolegend, 422201) at a concentration of 1 × 10^6^ cells/mL. Cells were then incubated with 0.5 µg/mL Annexin V-FITC and 10^−2^ µg/mL PI during 15 min. After incubation, 400 µL of the Annexin V binding buffer was added to cell suspensions. 60,000 events per sample were recorded using the FC 500 Beckman Coulter (Brea, CA, USA). Data were analyzed by the Kaluza Analysis Software (version 1.5.20365.16139).

### Proliferation assays

Cell cycle status was evaluated using Ki67-FITC and PI labeling and flow cytometry. 1 × 10^6^ cells were harvested 48 h post-transfection and fixed in 3 mL cold ethanol (70%) during 90 min. Cells were resuspended in 1 mL of cell staining buffer (Biolegend, 420201). 100 µL of cell suspensions were incubated with 0.06 µg/5 µL Ki67-FITC during 30 min. After incubation, cells were washed with cell staining buffer, resuspended in 500 µL of cell staining buffer and incubated with 10^−2^ µg/mL PI. 60,000 events per sample were analyzed by flow cytometry using the FC 500 Beckman Coulter (Brea, CA, USA). The cell cycle status was determined as previously described^34^.

### Cell migration and invasion assays

Transwell cell migration assays were performed using BD Falcon Cell Culture Inserts. MDA-MB-231 and BT549 cells expressing Src mutants were pre-incubated in serum-free medium (DMEM supplemented with 0.1% FBS) overnight. 25,000 cells resuspended in 200 µL of serum-free medium were placed in the insert and allowed to migrate for 24 h. The outer chamber was filled with 600 µL of medium containing 20% FBS or with 600 µL of serum-free medium (as negative control). After 24 h, non-migrating cells were removed with a cotton swab and migrating cells were fixed with methanol during 20 min and stained with crystal violet. For invasion assays, inserts were pre-coated with 100 µL matrigel (500 µg/mL) diluted in cold coating buffer (0.01 M Tris, 0.7% NaCl, pH 8) during 2 h. 25,000 cells were seeded in matrigel-coated inserts. Then, invasion was evaluated as described for migration assays. Five adjacent quadrants at the center of each membrane were imaged at ×40 magnification using the EVOS FL Auto 2 imaging system. Cells were counted (cell counts ranged from <10 to 800 per quadrant) and the mean number of cells/quadrant/membrane was determined.

### LC-MS/MS

Lysates of MDA-MB-231 cells expressing pcDNA or MLS-Src-HA were submitted to trypsin digestion and phospho-peptides enrichment using titanium dioxide (Pierce). Peptide samples were injected and separated by online reversed-phase (RP) nanoscale capillary liquid chromatography (nanoLC) and analyzed by electrospray mass spectrometry (ESI MS/MS). The experiments were performed with a Dionex UltiMate 3000 nanoRSLC chromatography system (Thermo Fisher Scientific / Dionex Softron GmbH, Germering, Germany) connected to an Orbitrap Fusion mass spectrometer (Thermo Fisher Scientific, San Jose, CA, USA). Peptides were eluted with a linear gradient of 5–40% B (A: 0.1% formic acid, B: 80% acetonitrile, 0.1% formic acid) for 120 min on a 75 u x 50 cm Acclaim Pepmap (ThermoFisher) column at 300 nl/min. Raw data were then analyzed by Mascot and Scaffold softwares to identify proteins and phospho-peptides using the database Uniprot Ref Homo sapiens (93675 entries). Quantitative analysis of phospho-peptide levels was performed using MaxQuant (version 1.6.0.16). Differences were considered significant between the two conditions when the *P*-value < 0.05 and the *Z*-score > 1.96 (i.e., outside the 95% confidence interval). Phospho-peptide enrichment, digestion and mass spectrometry were performed by the Proteomics Platform of the CHU de Quebec research center, Quebec city, Quebec, Canada.

### Levels of mtDNA

Total DNA was isolated from MDA-MB-231 cells using standard protocols. The relative amount of mtDNA was examined by real-time PCR as described^35^. Primers sequences for the mitochondrial gene *MT-ND5* (326 bp) were: forward: 5′-AGGCGC-TATCACCACTCTGTTCG-3′, reverse: 5′-AACCTGTGAGGAAAGGTATTCCTG-3′. Primers for the nuclear gene *CFTR* (460 bp) were: forward: 5′-ACAGAAGCGTCATCAAAGCA-3′, reverse: 5′-AGCTTACCCATAGAGGAAA-CATAA-3′. PCR was performed in 10 µl reaction volume containing 2 mM MgCl_2_, 0,5 µM each of the forward and reverse primers, 4 μl of DNA (5 ng) and 1 µl of master mixture (LightCycler-Fast Start DNA master SYBR Green I; Roche) containing TaqDNA polymerase, deoxynucleotide triphosphates and SYBR Green I. The same DNA dilutions were used for both nuclear DNA and mtDNA calibration curves with a standard DNA. The reaction was conducted as follows: an initial denaturing step at 95 °C for 3 min, followed by 50 cycles at 95 °C for 10 s, 60 °C for 30 s, with acquisition mode at segment 2. Samples were analyzed in triplicate and the number of copies of mtDNA was calculated as 2 × 2^∆Ct^, where ∆Ct = Ct_CFTR_ − Ct_ND5_, as described^36^.

### Statistical analyses

Statistical analyses were performed using GraphPad software (version 7.02). Quantitative analysis of phospho-peptide levels was performed using MaxQuant (version 1.6.0.16). Results are expressed as mean of independent data points ± s.e.m. Data were analyzed using Student T test, one-way (followed by Tukey’s post hoc test) or two-way ANOVA (followed by Dunnett’s post hoc test), as appropriate. Significance was assessed at *p* ≤ 0.05.

## Results

### Activity of mtSrc is higher in TNBC cells

**Fig. 1:**
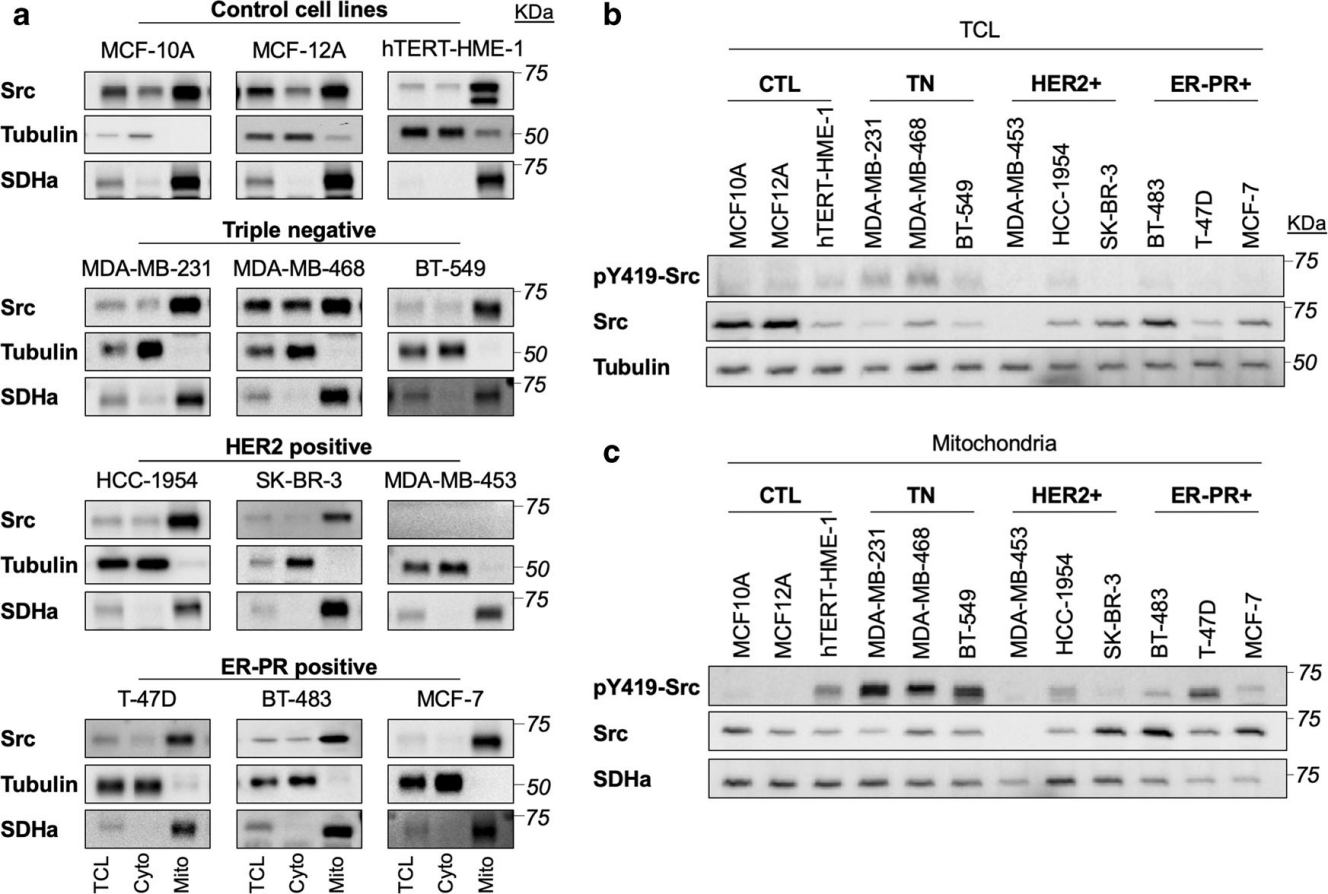
Intra-mitochondrial Src kinase is more active in triple negative breast cancer cells. **a** Representative immunoblottings (*n* = 4–6) of Src, the cytosolic protein tubulin and the mitochondrial protein succinate dehydrogenase a (SDHa) in different subcellular fractions derived from various breast cancer subtypes, showing that intra-mitochondrial Src kinase is present in most breast cancer and control cells. TCL: total cell lysate; Cyto: cytosolic fraction; Mito: mitochondrial fractions. **b** Representative immunoblottings (*n* = 4–6) of pY419-Src, total Src and tubulin in total cell lysate (TCL) obtained from breast cancer cell lines (see also Supplementary Fig. 1a, b for quantification); **c** Representative immunoblottings (*n* = 4–6) of pY419-Src, total Src and SDHa in mitochondrial-enriched fractions obtained from breast cancer cell lines, showing that mtSrc is more active in triple negative (TN) breast cancer cells (see also Supplementary Fig. 1a, b for quantification).

We first characterized the levels and activity of total Src and mtSrc in different subcellular fractions obtained from control breast epithelial cells, triple negative cells, HER2-positive cells and ER-PR-positive cells. Immunoblotting of the cytosolic marker tubulin and of the mitochondrial marker succinate dehydrogenase subunit a reveal the purity of the cytosolic and mitochondria-enriched fractions obtained by differential centrifugation (Fig. 1a). These immunodetection assays also revealed that Src is present both in cytosolic- and mitochondrial-enriched fractions in all of the cell lines tested (Fig. 1a). Only the HER2+ MDA-MB-453 cell line has low (if any) levels of Src (Fig. 1a, b), in accordance with previous works^37,38^. Direct comparison of total cell lysates by immunoblotting revealed no clear difference in total Src levels or activity specific to BC subtypes (Fig. 1b and Supplementary Fig. 1a, b). However, when Src levels and activity were compared among mitochondrial fractions, the activity of mtSrc appeared significantly higher in triple negative MDA-MB-231, MDA-MB-468 and BT549 cells than in other cell lines (Fig. 1c and Supplementary Fig. 1c, d). These results suggest that mtSrc signaling is involved in the specific phenotype of TNBC.

### MtSrc alters mitochondrial metabolism

**Fig. 2:**
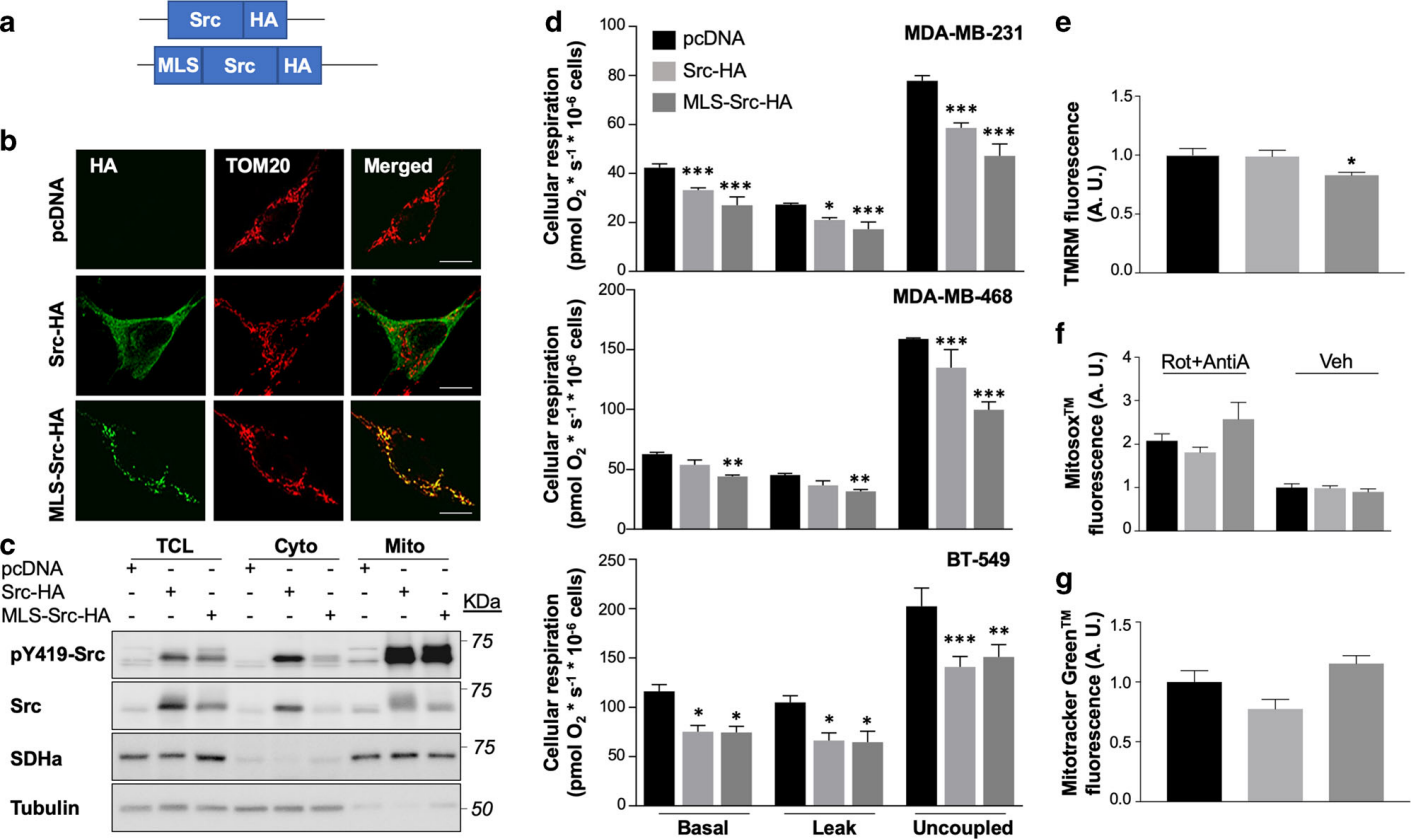
Overexpression of intra-mitochondrial Src blunts mitochondrial metabolism. **a** Schematic view of Src-HA and MLS-Src-HA constructs that were generated to discriminate the role of extra- vs intra-mitochondrial Src. **b** Representative immunofluorescence of HA-tag (green) and mitochondrial protein TOM20 (red) in MDA-MB-231 cells expressing empty vector (pcDNA), Src-HA or MLS-Src-HA (*n* = 4), showing that MLS-Src-HA is specifically targeted to mitochondria. Scale bar: 25 µm. **c** Representative immunoblottings (*n* = 5) of pY419-Src, Src, mitochondrial protein succinate dehydrogenase a (SDHa) and cytosolic protein tubulin in different subcellular fractions among various breast cancer subtypes, showing the different subcellular distribution of Src-HA and MLS-Src-HA. TCL: total cell lysate; Cyto: cytosolic fraction; Mito: mitochondrial fractions. **d** Different cellular respiratory states (basal, leak and uncoupled) of triple negative breast cancer cells MDA-MB-231, MDA-MB-468 and BT-549 expressing empty vector (pcDNA), Src-HA or MLS-Src-HA (*n* = 3), showing that expression of Src only in mitochondria is sufficient to decreases mitochondrial activity. *p ≤ 0.05, **p ≤ 0.01, ***p ≤ 0.001, according to two-way ANOVA followed by Dunnett’s multiple comparison test **e**–**g** Quantification of TMRM (**e**), MitoSox™ (**f**) and MitoTracker Green™ (**g**) fluorescence in MDA-MB-231 cells expressing empty vector (pcDNA), Src-HA or MLS-Src-HA (*n* = 5) showing that overexpression of mtSrc decreases mitochondrial membrane potential without affecting generation of superoxide or mitochondrial mass. See also Supplementary Fig. 2 for representative micrographs of TMRM, MitoSox™ and MitoTracker Green™ labeling. Rotenone (0.5 µM) and Antimycin A (2.5 µM) were added as positive control for MitoSox™ labeling. Rot: rotenone; AntiA: antimycin A. *p ≤ 0.05, **p ≤ 0.01, ***p ≤ 0.001 according to one-way ANOVA followed by Dunnett’s multiple comparison test. Data are presented as mean ± s.e.m.

To examine the specific role of mtSrc in TNBC cells, we generated a mutant of Src specifically targeted to mitochondria by fusing the mitochondrial leading sequence (MLS) of cytochrome c oxidase subunit VIII (amino acids 1–31) to the N-terminus of mouse Src (Fig. 2a). To discriminate between endogenous and ectopic Src, the tag hemagglutinin (HA) was added to the C-terminus of Src (Fig. 2a). Immunodetection of HA and mitochondrial protein TOM20 in MDA-MB-231 expressing control pcDNA vector, Src-HA or MLS-Src-HA by confocal microscopy revealed that Src-HA spreads throughout the cell, whereas MLS-Src-HA is specifically addressed to mitochondria (Fig. 2b). Immunoblottings confirmed that both constructs generate higher levels and higher activity of Src in specific subcellular compartments (Fig. 2c). These results indicate that Src-HA and MLS-Src-HA represent suitable tools to discriminate between extra- and intra-mitochondrial Src.

The functional impact of both Src mutants was then examined in TNBC cells. We first evaluated different respiratory states of intact MDA-MB-231, MDA-MB-468 and BT-549 cells. Overexpression of Src-HA significantly decreased most respiration states in TNBC cells (Fig. 2d). Overexpression of Src only in mitochondria was sufficient to inhibit cellular respiration (Fig. 2d). Similarly, mitochondrial membrane potential, an important parameter of OXPHOS, decreased upon expression of mtSrc, as indicated by lower TMRM fluorescence in MDA-MB-231 cells expressing MLS-Src-HA (Fig. 2e and Supplementary Fig. 2a). Alterations of OXPHOS induced by expression of MLS-Src-HA did not induced variation in superoxide production, as indicated by labeling of MitoSox™ in MDA-MB-231 expressing Src mutants (Fig. 2f and Supplementary Fig. 2b). Src mutants could decrease cellular respiration via reduction of global mitochondrial mass. We then examined whether Src mutants decrease cellular respiration via reduction of global mitochondrial mass using two approaches. MDA-MB-231 cells expressing Src mutants were labeled with MitoTracker Green™ which labels mitochondria independently of the mitochondrial membrane potential. Results obtained indicate that both Src mutants does not affect OXPHOS via reduction of mitochondrial mass (Fig. 2g and Supplementary Fig. 2c). Immunoblotting of a panel of mitochondrial proteins confirmed that mtSrc does not reduce levels of mitochondrial proteins (Supplementary Fig. 2d). Altogether, these results suggest that mtSrc induces OXPHOS defects via post-translational modification of mitochondrial protein(s).

**Fig. 3:**
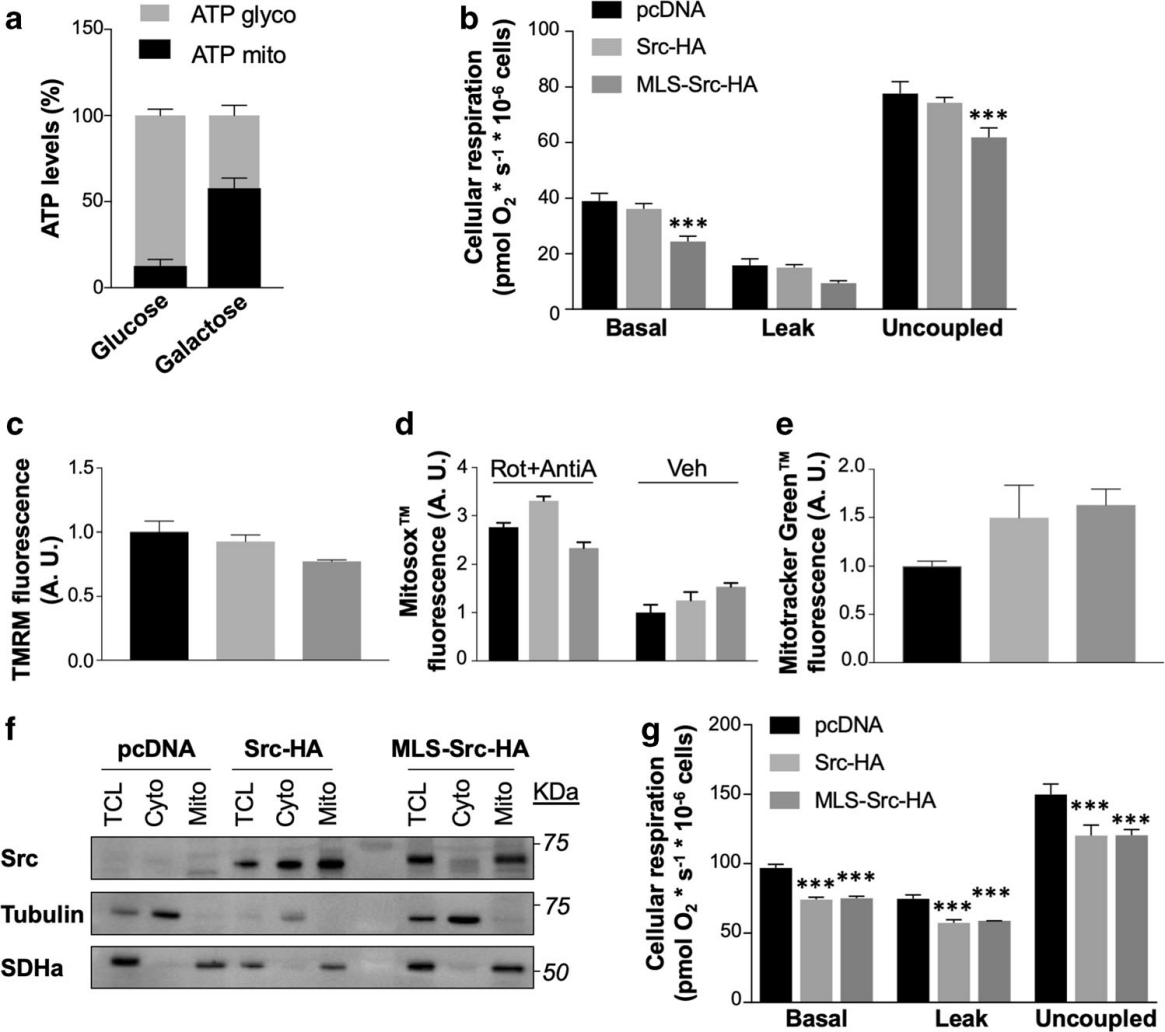
Mitochondrial alterations induced by intra-mitochondrial Src are not restricted to highly glycolytic triple negative cells. **a** ATP levels derived from glycolysis (ATP glyco) and mitochondrial metabolism (ATP mito) in MDA-MB-231 cells maintained in DMEM containing glucose or galactose (*n* = 5), showing that high glycolytic phenotype of cells cultured in DMEM with glucose can be modified to a more oxidative phenotype when glucose is replaced by galactose. **b** Different cellular respiratory states (basal, leak and uncoupled) of MDA-MB-231 cells expressing empty vector (pcDNA), Src-HA or MLS-Src-HA and maintained in DMEM containing galactose (*n* = 5), showing that mtSrc-dependent mitochondrial dysfunction decreases mitochondrial activity in pro-oxidative metabolism conditions. ***p ≤ 0.001, according to two-way ANOVA followed by Dunnett’s multiple comparison test. **c**–**e** Quantification of TMRM (**c**), MitoSox™ (**d**), and MitoTracker Green™ (**e**) fluorescence in MDA-MB-231 cells expressing empty vector (pcDNA), Src-HA or MLS-Src-HA and cultured in DMEM containing galactose (*n* = 3). See also Supplementary Fig. 3 for representative micrographs of TMRM, MitoSox™ and MitoTracker Green™ labeling. Rotenone (0.5 µM) and Antimycin A (2.5 µM) were added as positive control for MitoSox™ labeling. Rot: rotenone; AntiA: antimycin A. **f** Representative immunoblottings (*n* = 3) of Src, mitochondrial protein succinate dehydrogenase a (SDHa) and cytosolic protein tubulin in different subcellular fractions derived from HER2-positive MDA-MB-453 cells expressing empty vector (pcDNA), Src-HA or MLS-Src-HA. TCL: total cell lysate; Cyto: cytosolic fraction; Mito: mitochondrial fractions. **g** Different cellular respiratory states (basal, leak and uncoupled) of MDA-MB-453 cells expressing empty vector (pcDNA), Src-HA or MLS-Src-HA (*n* = 3), showing that mtSrc-dependent mitochondrial dysfunction is not restricted to triple negative breast cancer cells. Data are presented as mean ± s.e.m. ***p ≤ 0.001, according to two-way ANOVA followed by Dunnett’s multiple comparison test.

Cells grown in high-glucose media are usually highly glycolytic, as previously reported^26,39,40^. Indeed, ATP levels derived from mitochondrial metabolism are low compared to ATP generated from glycolysis in MDA-MB-231 cells grown in high-glucose DMEM (Fig. 3a). OXPHOS-dependent ATP levels can be increased when cells are grown in DMEM where glucose was replaced by galactose (Fig. 3a), as previously observed^26,41^ We thus examined the impact of mtSrc in DMEM containing galactose to determine whether mtSrc-dependent alterations of mitochondrial activity is restricted to highly glycolytic conditions. Overexpression of mtSrc had similar impact on cellular respiration, mitochondrial membrane potential, superoxide levels and mitochondrial mass in MDA-MB-231 cells grown in DMEM containing galactose (Fig. 3b–e and Supplementary Fig. 3) as compared to cells maintained in DMEM containing glucose (Fig. 2c–f and Supplementary Fig. 2a–c). Our findings suggest that the mtSrc-dependent modulation of mitochondrial metabolism is not specific to TNBC glycolytic phenotype. Expression of Src mutants in MDA-MB-453 cells (Fig. 3f), which have low levels of endogenous Src (Fig. 1 and Supplementary 1) also inhibited cellular respiration (Fig. 3g). These findings indicate that mtSrc-dependent regulation of cellular respiration is not restricted to TNBC cells and/or cells with high endogenous levels of (mt)Src.

### MtSrc modulates cellular viability and proliferation

**Fig. 4:**
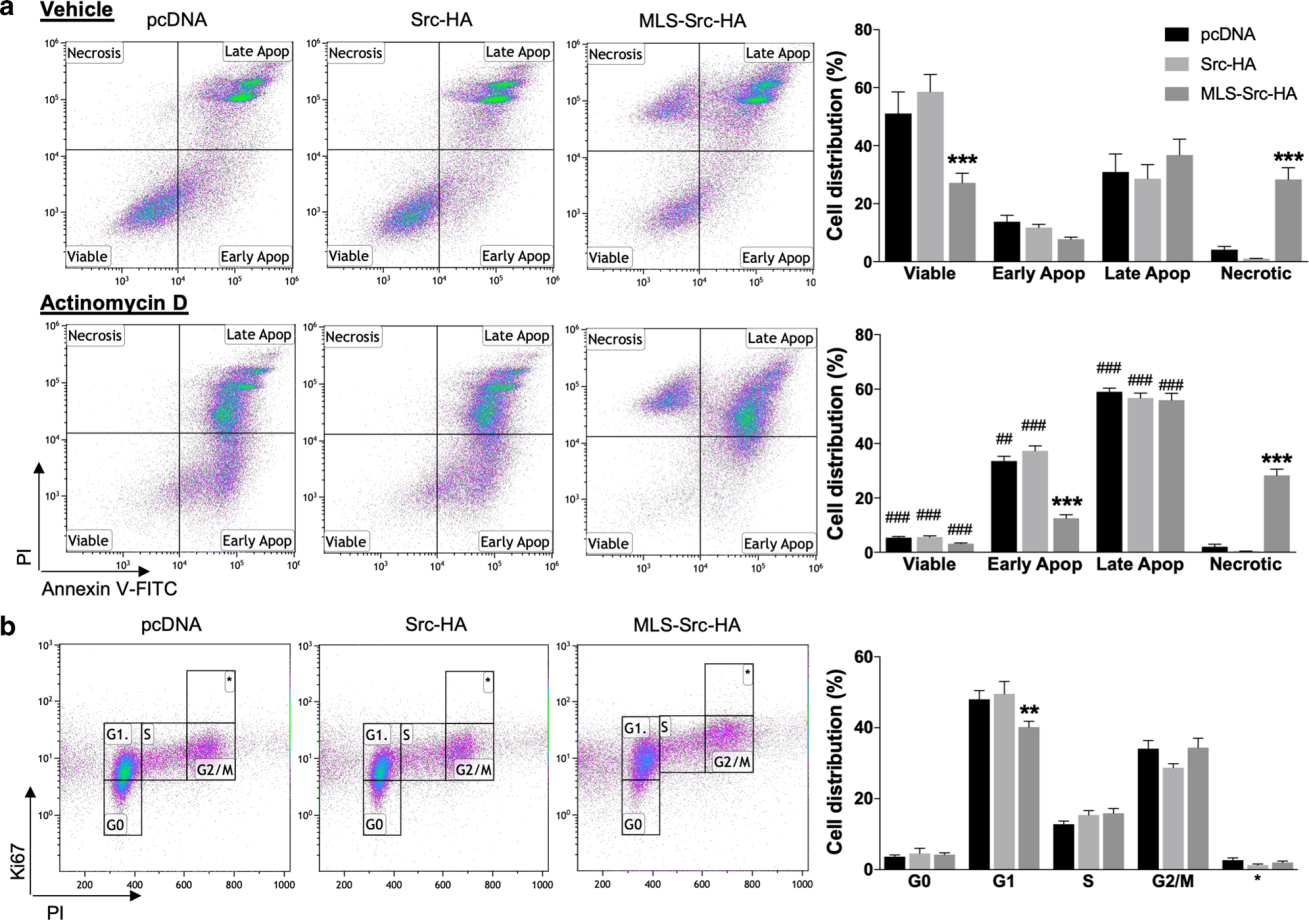
Intra-mitochondrial Src has specific effects on viability and cell cycle in MDA-MB-231 cells. **a** Flow cytometry analyses (left) and quantification (right) of MDA-MB-231 cells expressing empty vector (pcDNA), Src-HA or MLS-Src-HA, treated with vehicle or Actinomycin D (5 µM, 48 h) and stained with PI and Annexin V-FITC (*n* = 6), showing that overexpression of mtSrc in MDA-MB-231 decreases the number of viable cells and block early apoptosis upon treatment with Act. D. **b** Flow cytometry analyses (left) and quantification (right) of MDA-MB-231 cells expressing empty vector (pcDNA), Src-HA or MLS-Src-HA labeled with Ki67 and PI (*n* = 7–13), indicating that overexpression of mtSrc decreases the number of cells in G1 phase. Data are presented as mean ± s.e.m. For A, ***p ≤ 0.001 as compared to pcDNA; ##*p* ≤ 0.01, ###*p* ≤ 0.001 as compared to vehicle (veh), according to two-way ANOVA followed by Dunnett’s multiple comparison test. For **b**, **p ≤ 0.01 as compared to pcDNA according to two-way ANOVA followed by Dunnett’s multiple comparison test.

Mitochondrial functions are involved in the neoplasic phenotype of cancer cells^5^. We thus hypothesized that mtSrc-dependent alterations of mitochondrial metabolism could affect the phenotype of TNBC cells. To address this, we examined how mtSrc impacts on apoptotic, proliferative, invasion and migration capacities of MDA-MB-231 cells. MDA-MB-231 cells expressing control vector, Src-HA or MLS-Src-HA grown in high-glucose or galactose media were labeled with Annexin V-FITC and PI to characterize the impact of mtSrc on cellular viability. Expression of Src-HA had no effect on cell death whereas expression of MLS-Src-HA resulted in significant reduction of viable cells and increase in necrotic cells grown in high-glucose (Fig. 4a). When glucose was replaced by galactose in culture media, overexpression of mtSrc decreased the number of viable cells but increased late apoptotic cells (Supplementary Fig. 4a). MDA-MB-231 cells were then treated with the apoptotic inducer actinomycin D (Act. D) to evaluate the role of mtSrc during pro-apoptotic conditions. Expression of Src-HA had no impact on viability of Act. D-treated cells compared to cells expressing control vectors (Fig. 4a and Supplementary Fig. 4a). However, expression of MLS-Src-HA significantly reduced early apoptosis and increased necrosis in cells treated with Act. D, independently of their metabolic phenotype (Fig. 4a and Supplementary Fig. 4a). Expression of MLS-Src-HA also blocked the increase of early apoptotic cells induced by Act. D (Fig. 4a and Supplementary Fig. 4a). Overall, these findings indicate that mtSrc activity modulates cellular viability and apoptosis per se and partly blocks the sensitivity of MDA-MB-231 cells to the apoptotic inducer Act. D.

**Fig. 5:**
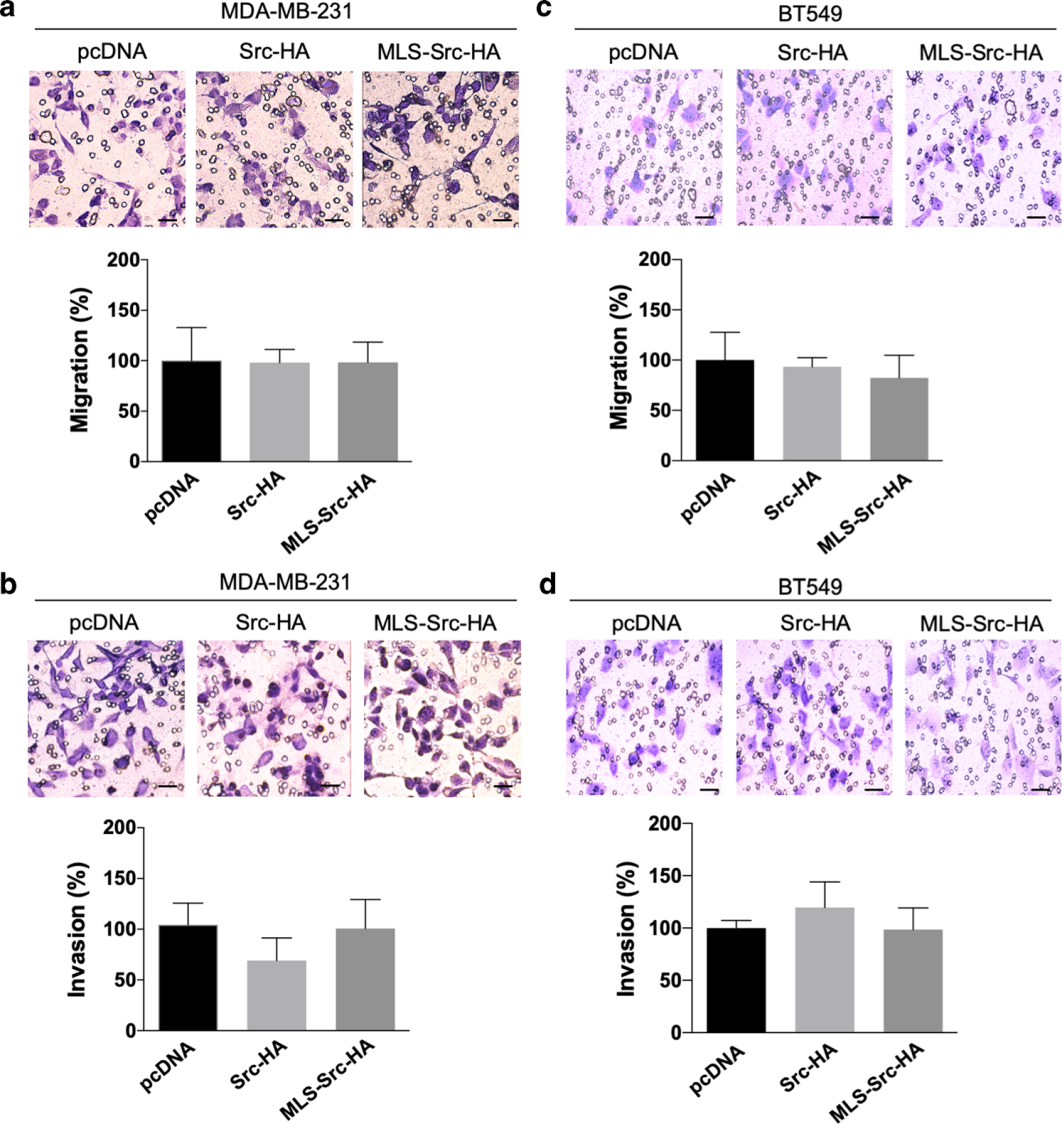
Migration and invasion capacities upon expression of (mt)Src in MDA-MB-231 and BT549 cells. **a** Representative micrographs and quantification of transwell assays without matrigel to evaluate migration of MDA-MB-231 cells expressing empty vector (pcDNA), Src-HA or MLS-Src-HA (*n* = 5). **b** Representative micrographs and quantification of transwell assays with matrigel to evaluate invasion capacities of MDA-MB-231 cells expressing empty vector (pcDNA), Src-HA or MLS-Src-HA (*n* = 5). **c** Representative micrographs and quantification of transwell assays without matrigel to evaluate migration of BT549 cells expressing pcDNA, Src-HA or MLS-Src-HA (*n* = 5). **d** Representative micrographs and quantification of transwell assays with matrigel to evaluate invasion capacities of BT549 cells expressing empty vector pcDNA, Src-HA or MLS-Src-HA (*n* = 5). Scale bar: 50 µm. Data are presented as mean ± s.e.m. Data were analyzed by one-way ANOVA.

Cells expressing Src mutants were labeled with PI and the proliferation marker Ki67 to examine the impact of mtSrc on cell cycle and cellular proliferation, as described^34^. In both metabolic conditions (high-glucose vs galactose DMEM), expression of Src-HA had no effect on cell cycle status. In high-glucose DMEM, expression of mtSrc significantly decreased numbers of cells in G1 phase (Fig. 4b). When cells were maintained in galactose DMEM, the number of cells in G1 phase was significantly lower whereas number of cells in G2/M was increased (Supplementary Fig. 4b), indicating that mtSrc shortens the cell cycle and accelerates proliferation. We then examined the impact of Src overexpression in migration and invasion capacities of TNBC cells. The findings obtained suggest that (mt)Src has no impact on migration in MDA-MB-231 and BT549 cells (Fig. 5a, c and Supplementary Fig. 5a, c). However, invasiveness of MDA-MB-231 and BT549 cells expressing either Src-HA and MLS-Src-HA was increased when cells were maintained in galactose media (Fig. 5b, d and Supplementary Fig. 5b, d), indicating that mtSrc increases invasion capacities in pro-OXPHOS conditions. Overall, our findings indicate that the mtSrc-dependent remodeling of metabolism is crucial in the development of malignancy of TNBC.

### MtSrc does not impact on mitochondria via COXII and EGF signaling

**Fig. 6:**
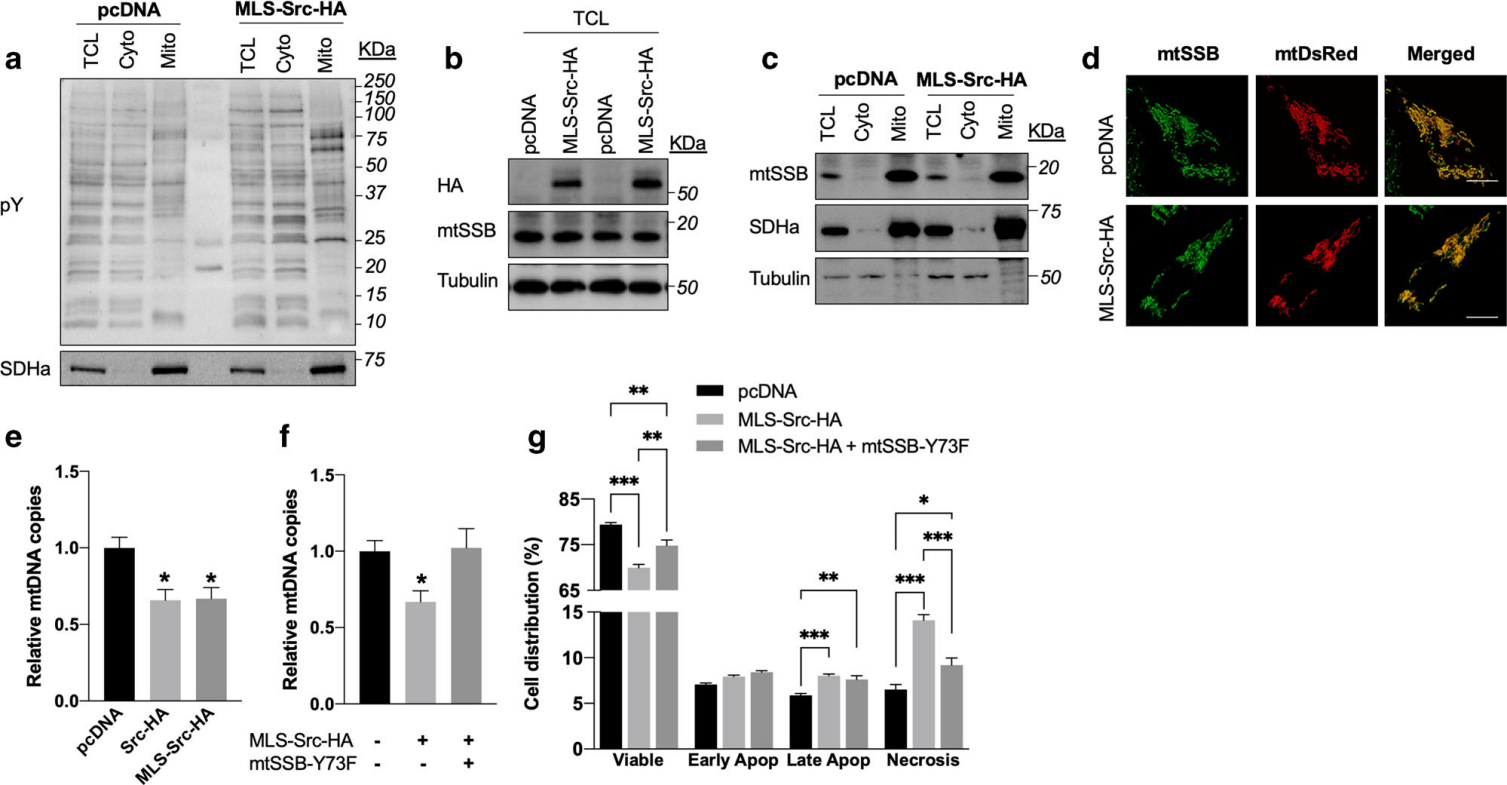
Intra-mitochondrial Src kinase decreases mtDNA levels via phosphorylation of mitochondrial single-stranded DNA-binding protein. **a** Representative immunoblottings (*n* = 5) of tyrosine-phosphorylation (pY) and the mitochondrial protein SDHa among subcellular fractions derived from MDA-MB-231 cells expressing empty vector (pcDNA) or MLS-Src-HA. Note the increased tyrosine-phosphorylation among mitochondrial proteins upon overexpression of mtSrc. **b** Representative immunoblottings of mitochondrial single-stranded DNA-binding protein (mtSSB) and tubulin in total cell lysates (TCL) derived from MDA-MB-231 cells expressing empty vector (pcDNA) or MLS-Src-HA (n = 3), indicating that overexpression of mtSrc does not change levels of mtSSB. **c**, **d** Representative immunoblottings (*n* = 3) of mtSSB, SDHa and tubulin among subcellular fractions of MDA-MB-231 cells expressing pcDNA or MLS-Src-HA (**c**), and representative micrographs (*n* = 3) of MDA-MB-231 cells co-expressing a mitochondria-targeted DsRed and pcDNA or MLS-Src-HA (D), indicating that subcellular localization of mtSSB does not change upon overexpression of mtSrc. TCL: total cell lysate; Cyto: cytosolic fraction; Mito: mitochondrial fraction. Scale bar: 25 μm. **e** Relative levels of mtDNA copies in MDA-MB-231 cells expressing empty vector, Src-HA or MLS-Src-HA (*n* = 4–5), indicating that overexpression of mtSrc reduces the amount of mtDNA. **f** Relative levels of mtDNA copies in MDA-MB-231 cells expressing pcDNA, MLS-Src-HA or co-expressing MLS-Src-HA with the phospho-deficient mtSSB-Y73F (*n* = 4–5), indicating that overexpression of mtSrc reduces the amount of mtDNA via phosphorylation of mtSSB-Y73. **g** Quantification of flow cytometry analyses of MDA-MB-231 cells expressing empty vector, MLS-Src-HA, or co-expressing mtSrc and mtSSB-Y73F stained with PI and Annexin V-FITC (*n* = 6), showing that mtSrc impacts on viability via phosphorylation of mtSSB-Y73 (see also Supplementary Fig. 6e). Data are presented as mean ± s.e.m. For E and F, *p ≤ 0.05 as compared to pcDNA, according to one-way ANOVA followed by Tukey’s multiple comparison test. Data presented in G were analyzed by two-way ANOVA followed by Tukey’s multiple comparison test (*p ≤ 0.05, **p ≤ 0.01, ***p ≤ 0.001).

Src targets several mitochondrial proteins^30^. Conversely, expression of MLS-Src-HA in MDA-MB-231 cells increased tyrosine-phosphorylation of several mitochondrial proteins (Fig. 6a). The subunit II of cytochrome *c* oxidase (COXII) was the first protein identified as a target of mtSrc^25^. Later, it was shown that COXII can be phosphorylated by mtSrc upon treatment with epidermal growth factor (EGF)^27^. Considering these findings, we examined whether the mtSrc-dependent regulation of mitochondrial metabolism observed here is linked to EGF- and Src-dependent phosphorylation of COXII. Immunoprecipitation of COXII revealed that overexpression of mtSrc does not increase tyrosine-phosphorylation of COXII in MDA-MB-231 cells (Supplementary Fig. 6a). Also, no variation in tyrosine-phosphorylation of COXII after treatment with EGF was observed (Supplementary Fig. 6a). Activation and translocation of Src into mitochondria were previously shown to be increased upon EGF treatment^27^. No variation in mtSrc activity and translocation of Src into mitochondria upon treatment with EGF was however observed (Supplementary Fig. 6b, c). EGF-dependent phosphorylation of COXII was shown to lower ATP levels in MDA-MB-231 cells^27^. EGF had however no impact on cellular respiration of MDA-MB-231 cells (Supplementary Fig. 6d). Overall, these results suggest that mtSrc and EGF signaling are independent in MDA-MB-231.

### MtSrc increases phosphorylation of mtSSB and reduces mtDNA levels

To identify the target(s) of mtSrc, we quantitatively analyzed phospho-proteins in MDA-MB-231 cells expressing either empty vector or MLS-Src-HA by LC-MS/MS. As shown in Table 1, 18 phospho-peptides were significantly more abundant upon overexpression of mtSrc. Gene ontology enrichment analyses (performed using Gene Ontology Unifying Biology online tool, http://geneontology.org/) revealed an over-representation of genes involved in cadherin binding (five genes out of 18 [*TBC1D2*, *MICALL1*, *SCRIB*, *PLEC*, *DLC1*], representing a 17,5-fold enrichment as compared to the total human genome, *p* < 0,001). Among the 18 proteins identified, only the mitochondrial single-stranded DNA-binding protein (mtSSB) is located inside the organelle and thus proximal to mtSrc, suggesting it is directly phosphorylated by mtSrc. Phosphorylated mtSSB was 34-fold more abundant following expression of MLS-Src-HA (Table 1). Moreover, mtSSB was the only phospho-peptide for which a tyrosine residue was identified as the putative phosphorylation site (i.e., Y73, see Supplementary Table 1). We therefore examined levels, subcellular localization and activity of mtSSB upon overexpression of mtSrc in MDA-MB-231. We first observed that expression of MLS-Src-HA does not change the total levels of mtSSB (Fig. 6b), indicating that the higher level of phosphorylation of mtSSB upon overexpression of mtSrc is not due to higher levels of mtSSB per se. Immunofluorescence and immunoblotting of mtSSB in subcellular compartments revealed that overexpression of mtSrc and phosphorylation of mtSSB does not change the intra-mitochondrial localization of the protein (Fig. 6c, d). Since mtSSB is required for the maintenance of mtDNA^42,43^, we measured levels of mtDNA as an index of mtSSB activity. Strikingly, we observed that overexpression of (mt)Src results in lower mtDNA levels (Fig. 6e), suggesting that mtSrc-dependent phosphorylation of mtSSB reduces its replication activity. To address this, we generated a phospho-deficient mutant of mtSSB-Y73 (i.e., mtSSB-Y73F). Then, mtDNA levels were compared among MDA-MB-231 cells expressing control vector, MLS-Src-HA and MLS-Src-HA with mtSSB-Y73F. Strikingly, co-expression of mtSSB phospho-deficient and MLS-Src-HA completely prevented the reduction of mtDNA levels induced by mtSrc expression (Fig. 6f). Similarly, co-expression of the same constructs prevented the changes in number of viable cells and necrotic cells induced by over-expression of mtSrc (Fig. 6g and Supplementary Fig. 6e), indicating that mtSrc impacts on TNBC cells via phosphorylation of mtSSB-Y73.

**Table 1:** Phospho-proteins identified by LC-MS/MS upon expression of mtSrc.

Protein name	Gene name	Ratio	*P* value	GO molecular function
Single-stranded DNA-binding protein, mitochondrial	*SSBP1*	34.40	*p* < 0.05	DNA and RNA binding
Probable ATP-dependent RNA helicase DDX47	*DDX47*	17.57	*p* < 0.01	ATP and RNA binding
Serine/arginine-rich splicing factor 1	*SRSF1*	13.54	*p* < 0.05	RNA binding
Pumilio homolog 2	*PUM2*	11.62	*p* < 0.05	mRNA binding
TBC1 domain family member 2A	*TBC1D2*	9.90	*p* < 0.05	Cadherin binding
Caskin-2	*CASKIN2*	8.92	*p* < 0.001	No biological data available
Probable ATP-dependent RNA helicase DDX20	*DDX20*	8.91	*p* < 0.01	ATP and DNA binding
MICAL-like protein 1	*MICALL1*	8.51	*p* < 0.05	Cadherin binding
Protein TANC2	*TANC2*	7.79	*p* < 0.01	No biological data available
TMEM63B	*TMEM63B*	7.06	*p* < 0.05	Calcium channel activity
Protein scribble homolog	*SCRIB*	6.51	*p* < 0.05	Cadherin binding
Cytochrome b reductase 1	*CYBRD1*	6.17	*p* < 0.05	Oxidoreductase activity
Ankyrin repeat and IBR domain-containing protein 1	*ANKIB1*	5.89	*p* < 0.05	Ubiquitin-ligase activity
Plectin	*PLEC*	5.55	*p* < 0.05	Cadherin binding
Rho guanine nucleotide exchange factor 2	*ARHGEF2*	5.35	*p* < 0.01	Rho-GTPase activity
Eukaryotic translation initiation factor 4 gamma 2	*EIF4G2*	5.25	*p* < 0.01	mRNA binding
Insulin receptor substrate 2	*IRS2*	4.97	*p* < 0.05	Insulin receptor binding
Rho GTPase-activating protein 7	*DLC1*	4.92	*p* < 0.05	Rho-GTPase activity

Phospho-peptides were characterized from lysates derived from MDA-MB-231 cells expressing empty vector (pcDNA) or MLS-Src-HA. Ratio represents the number of phospho-peptides observed in cells expressing MLS-Src-HA to cells expressing pcDNA. Note that only phospho-peptides significantly more abundant in cells expressing MLS-Src-HA are shown. Go molecular functions were obtained from Uniprot (https://www.uniprot.org/). See Supplementary Table 1 for the complete dataset.

Supplemental Table 1. Complete dataset for LC-MS/MS analyses of phospho-peptides in MDA-MB-231 cells expressing pcDNA and MLS-Src-HA

Overall, our results demonstrate that higher activation of mtSrc increases phosphorylation of mtSSB to reduce mtDNA levels, inducing OXPHOS deficits, lower viability, shorter cell cycle and increased invasiveness in TNBC cells.

## Discussion

Alterations in mitochondrial functions and Src kinase activity are involved in the physiopathology of cancer. The role of intra-mitochondrial Src in this context remains however scantly studied. The aim of this work was to characterize the role of mtSrc in the metabolic and neoplasic phenotype of BC cells. Our findings indicate that mtSrc is more active in TNBC and that overexpression of mtSrc alters the metabolic and neoplasic behavior of TNBC cells via phosphorylation of mtSSB. This work therefore illustrates that mtSrc links alterations in mitochondrial metabolism to the aggressive phenotype of TNBC cells.

The present work identified a new target for mtSrc since its overexpression increases phosphorylation of mtSSB-Y73. Phosphorylation of mtSSB-Y73 has been observed in various type of cancer, including BC^44–50^. This protein is a key component of the mitochondrial replisome and is required for the mtDNA maintenance and replication. MtSSB binds and organizes template DNA, leading to stimulation of the mtDNA helicase Twinkle and the polymerase γ^42,43^. Reduction in mtDNA copy number and the maintenance of mtDNA levels by mtSSB has been associated with metabolic reprogramming and aggressive features such as increased metastatic potential and chemoresistance in various types of cancer^51,52^. Knock-down of mtSSB decreases mtDNA copy number and proliferation in different types of cancer cells, including Hela and 143B^53,54^. Silencing of mtSSB in human lung cancer cell line H1299 results in mitochondrial dysfunction and sensitization to ionizing radiation^55^. Interestingly, analysis of the Cancer Genome Atlas breast cancer dataset revealed that TN tumors have decreased mtDNA copy numbers^56^, whereas low mtSSB expression correlates with poor clinical outcomes^57^. In BC, silencing mtSSB decreases mtDNA levels and mitochondrial membrane potential, which trigger mitochondrial retrograde signaling mediated by calcineurin A resulting in increased epithelial-to-mesenchymal transition and metastatic potential in TNBC MDA-MB-231 and MDA-MB-468 cells^57^. Here, we observed that mtSrc increases tyrosine-phosphorylation of mtSSB-Y73F and reduces mtDNA levels. We therefore propose that mtSrc modulates metabolic and neoplasic phenotype of TNBC cells (at least partly) via phosphorylation of mtSSB. Our findings also demonstrate that extra- and intra-mitochondrial Src have different functional impacts on TNBC cells. Indeed, only MLS-Src-HA altered viability and cell cycle status although expression of Src-HA and MLS-Src-HA generated similar levels of ectopic mtSrc. It is thus possible that targets of extra-mitochondrial Src counteract some of the functionnal impact of mtSrc-mediated phosphorylation of mtSSB.

The present study suggests that mtSrc is central in the specific metabolic phenotype of TNBC cells. These cells exhibit higher glucose uptake and lactate production as well as lower mitochondrial metabolism than other BC subtypes^7,56^. Increased capacities of TNBC cells for metastasis is also linked to defects in mitochondrial dynamics^10^. Lower levels of mitophagy-related BNip3 is commonly found in TBNC, leading to defective mitochondria and higher metastatic potential^58^. Mitochondrial dysfunctions induced by lower mtDNA levels and/or altered OXPHOS can lead to Ca^2+^-dependent mitochondrial retrograde signaling, and activation of different pathways, such as nuclear translocation of NF-kB, important for metabolic reprogramming, induction of epithelial-to-mesenchymal transition, increased metastasis, and apoptosis resistance^59^. Rescuing mitochondrial activity could therefore represent a potent therapeutic strategy for TNBC. Increased complex I activity inhibits tumor growth and metastasis in TNBC^60^. Reactivation of mitochondrial metabolism by expression of mitochondrial pyruvate carrier also impairs the ability of different cancer cells to grow^61–63^. Strikingly, Src induces phosphorylation and inhibition of pyruvate dehydrogenase^64^, the enzyme responsible for the conversion of pyruvate into acetyl-CoA. Our results suggest that mtSrc-dependent reduction of mtDNA levels lead to OXPHOS deficiency and a more aggressive phenotype. Inhibitors specific for mtSrc could therefore restore mitochondrial activity in TNBC cells, and ultimately stop malignancy. It will be important to characterize the ability of known inhibitors and/or activators of Src to target mtSrc, and examine their potency towards mitochondrial functions and tumorigenesis. The overall metabolic phenotype of TNBC cells also appears important to predict the effects of (mt)Src activation. The present study shows that overexpression of mtSrc is able to increase invasion of MDA-MB-231 cells when they are maintained in a culture media that favors OXPHOS metabolism. Therefore, inhibition of Src could be even more beneficial in tumors using oxidative metabolism as compared to glycolytic tumors.

LC-MS/MS analyses showed that several proteins involved in cell-cell adhesion and invasion are also more phosphorylated upon overexpression of mtSrc. Although the role of these phosphorylation events was not characterized, they are indicative of global changes leading to a more invasive and aggressive phenotype. The protein scribble localizes to cell-cell junctions and regulates several signaling pathways involved in cell migration, whereas mislocalization of scribble promotes cell transformation and neoplasic growth in breast cancer^65,66^. TBC1 domain family member 2A, also known as Armus, is involved in E-cadherin degradation during cell dispersion^67^. MICAL-like protein 1 modulates invasion capacities via oxidative stress in breast cancer cells^68^. Plectin is a large intracellular protein which links cell-cell junctions, such as hemidesmosomes, to the cytoskeleton. Expression of plectin appears higher in cancer tissue and plectin-deficiency can result in higher migration^69,70^. The Rho guanine nucleotide exchange factor 2 (ARHGEF2, also known as GEF-H1) is an oncoprotein that activates RhoA and is involved in cytoskeleton organization, cellular motility, invasion and proliferation^71^. Interestingly, expression of ARHGEF2 accelerates breast cancer invasion and metastasis^72^. Similarly, active Rho GTPase activating protein 7 induces cell detachment through cytoskeletal reorganization and increases cell migration^73^. Altogether, our findings suggest that mtSrc links alterations of mitochondrial physiology and invasiveness in TNBC cells.

In conclusion, the present work demonstrates that Src localized inside mitochondria has specific functional consequences in BC. We observed that mtSrc makes TNBC more aggressive with alterations in mitochondrial metabolism and higher invasion. This work also identified a new target for mtSrc, i.e., mtSSB, which was already shown as a reliable prognostic marker in TNBC. Therefore, we propose that targeting mtSrc or its interactors should be considered in the generation of better therapeutic strategies for BC.

## Supplementary information

Supplemental Figure 1

Supplemental Figure 2

Supplemental Figure 3

Supplemental Figure 4

Supplemental Figure 5

Supplemental Figure 6

Supplemental Table 1

Author Contribution

Reproducibility Checklist
