# Supplementary figures and images for: Intramitochondrial Src kinase links mitochondrial dysfunctions and aggressiveness of breast cancer cells
Source: Cell Death Dis. 2019 Dec 9;10(12):940. doi: 10.1038/s41419-019-2134-8 (PMC6901437; doi:10.1038/s41419-019-2134-8)

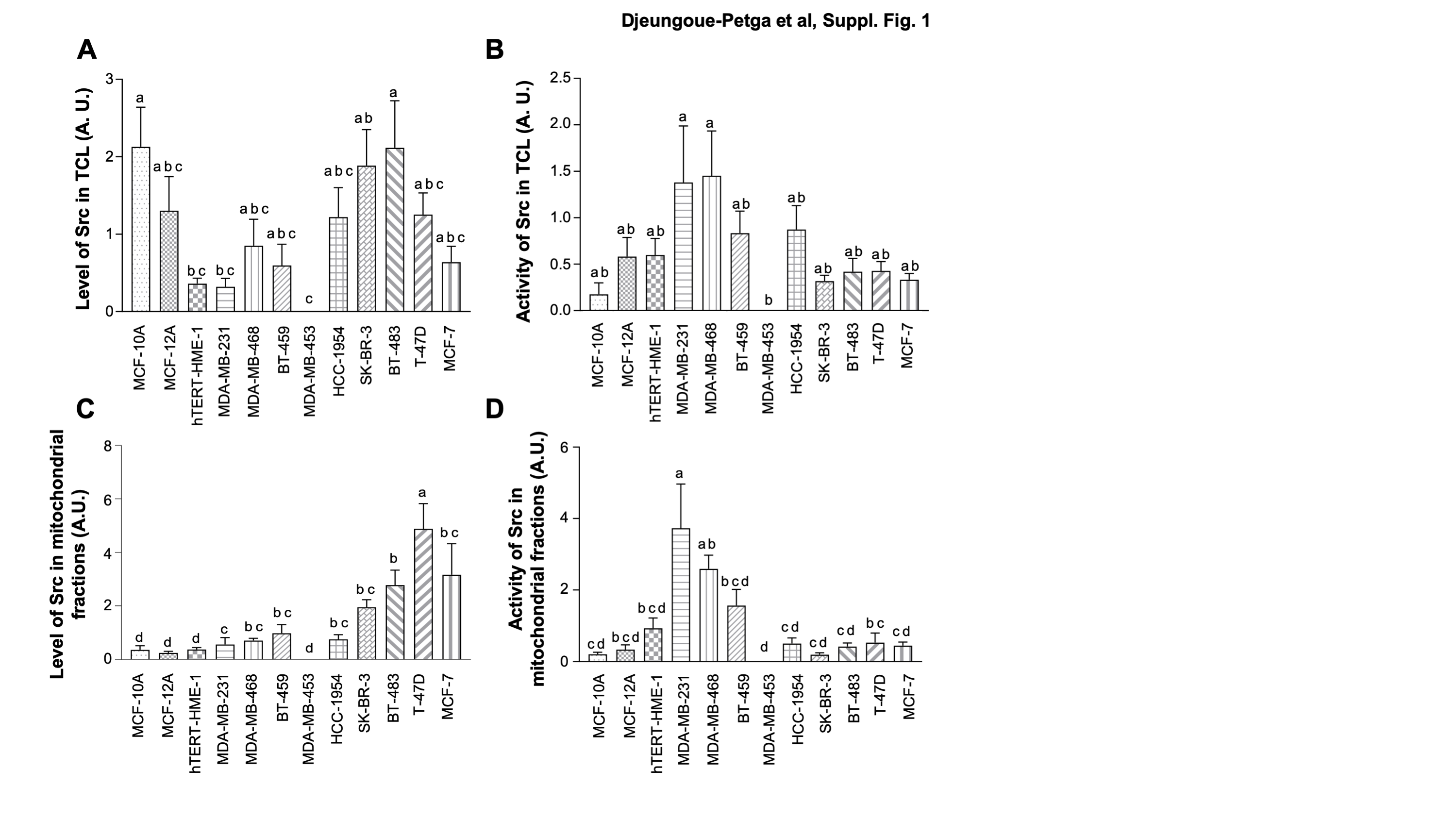

Supplement: Supplementary file 1 — Supplemental Figure 1 [file 41419_2019_2134_MOESM1_ESM.tif]

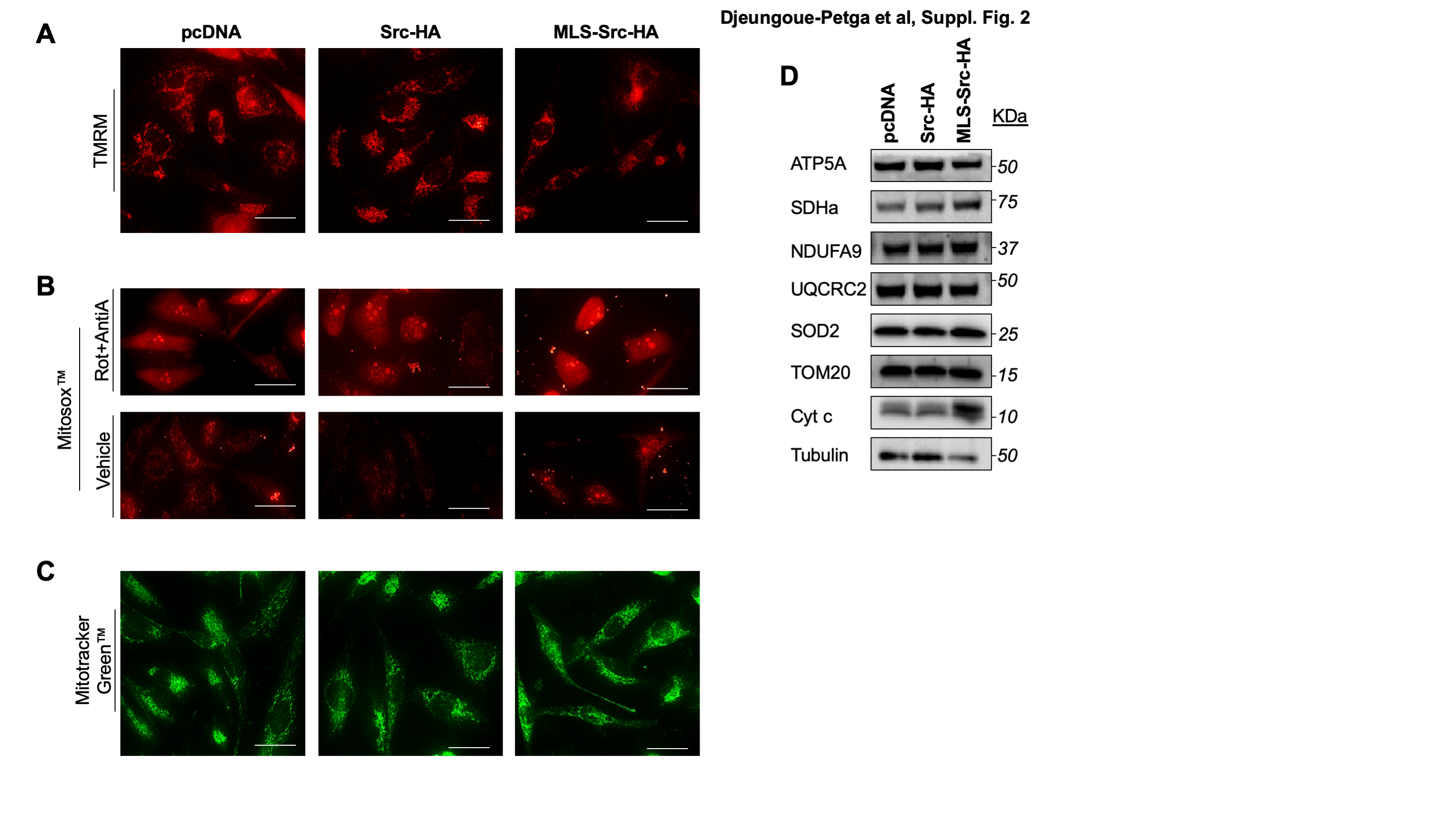

Supplement: Supplementary file 2 — Supplemental Figure 2 [file 41419_2019_2134_MOESM2_ESM.tif]

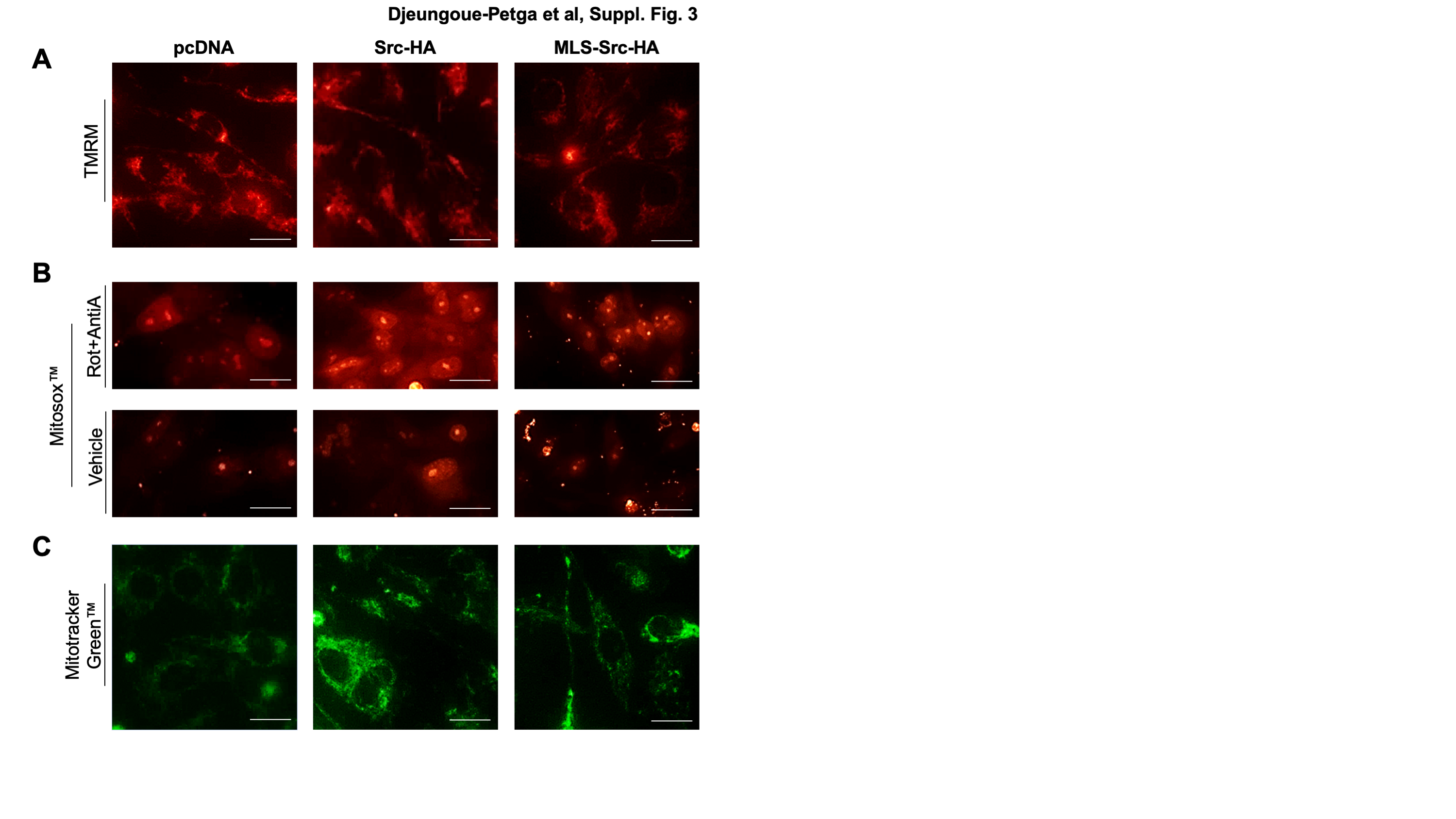

Supplement: Supplementary file 3 — Supplemental Figure 3 [file 41419_2019_2134_MOESM3_ESM.tif]

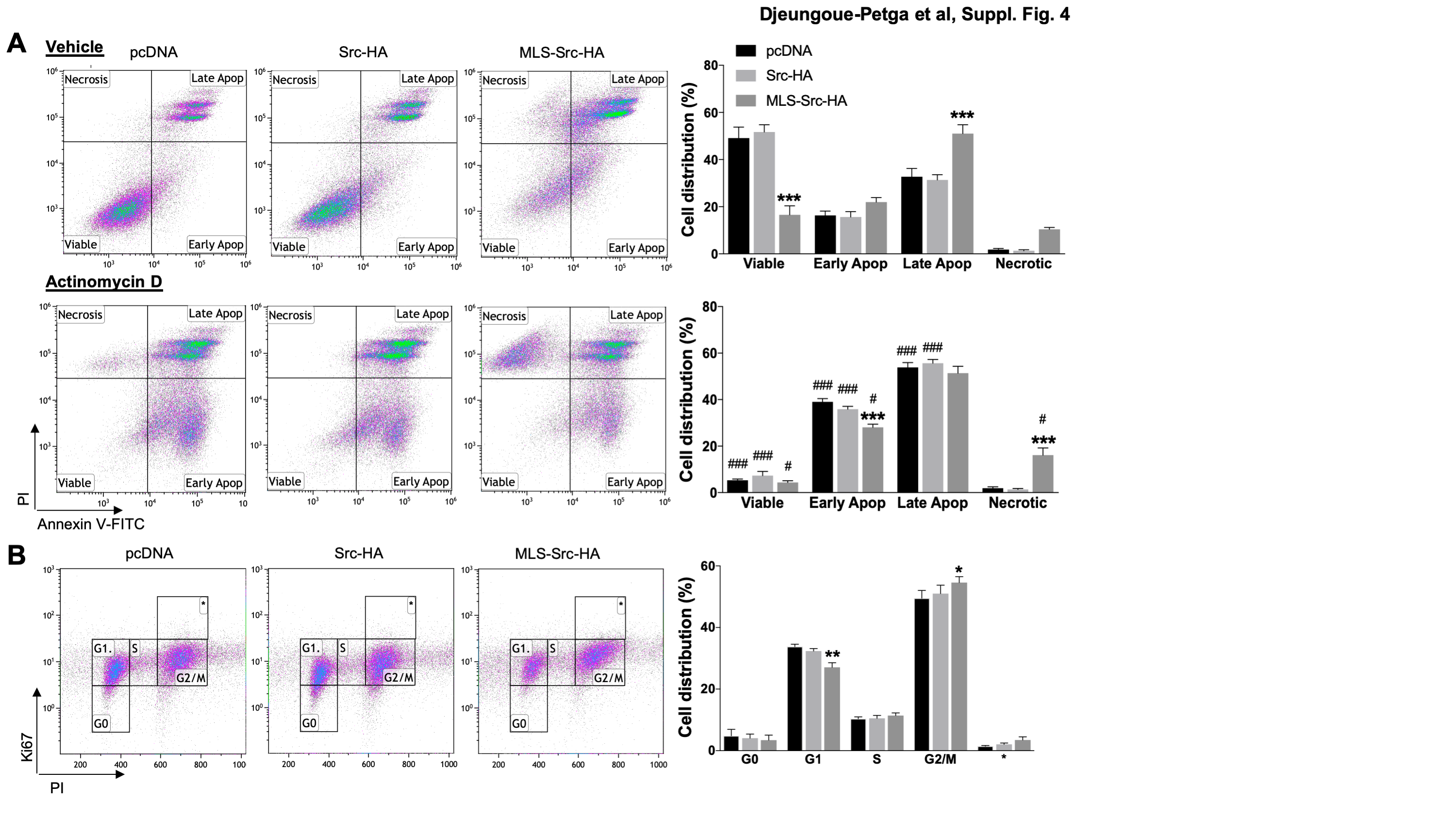

Supplement: Supplementary file 4 — Supplemental Figure 4 [file 41419_2019_2134_MOESM4_ESM.tif]

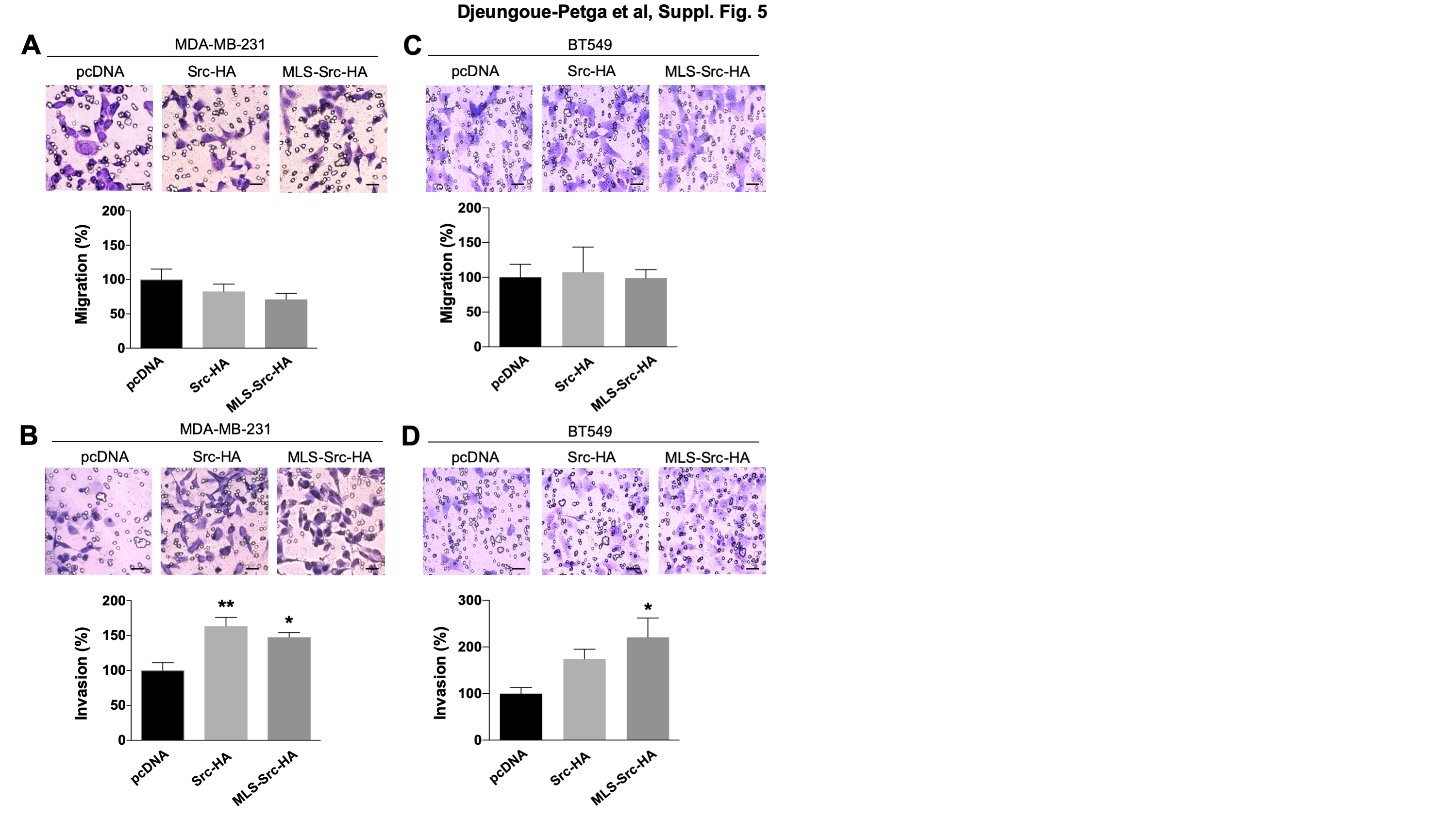

Supplement: Supplementary file 5 — Supplemental Figure 5 [file 41419_2019_2134_MOESM5_ESM.tif]

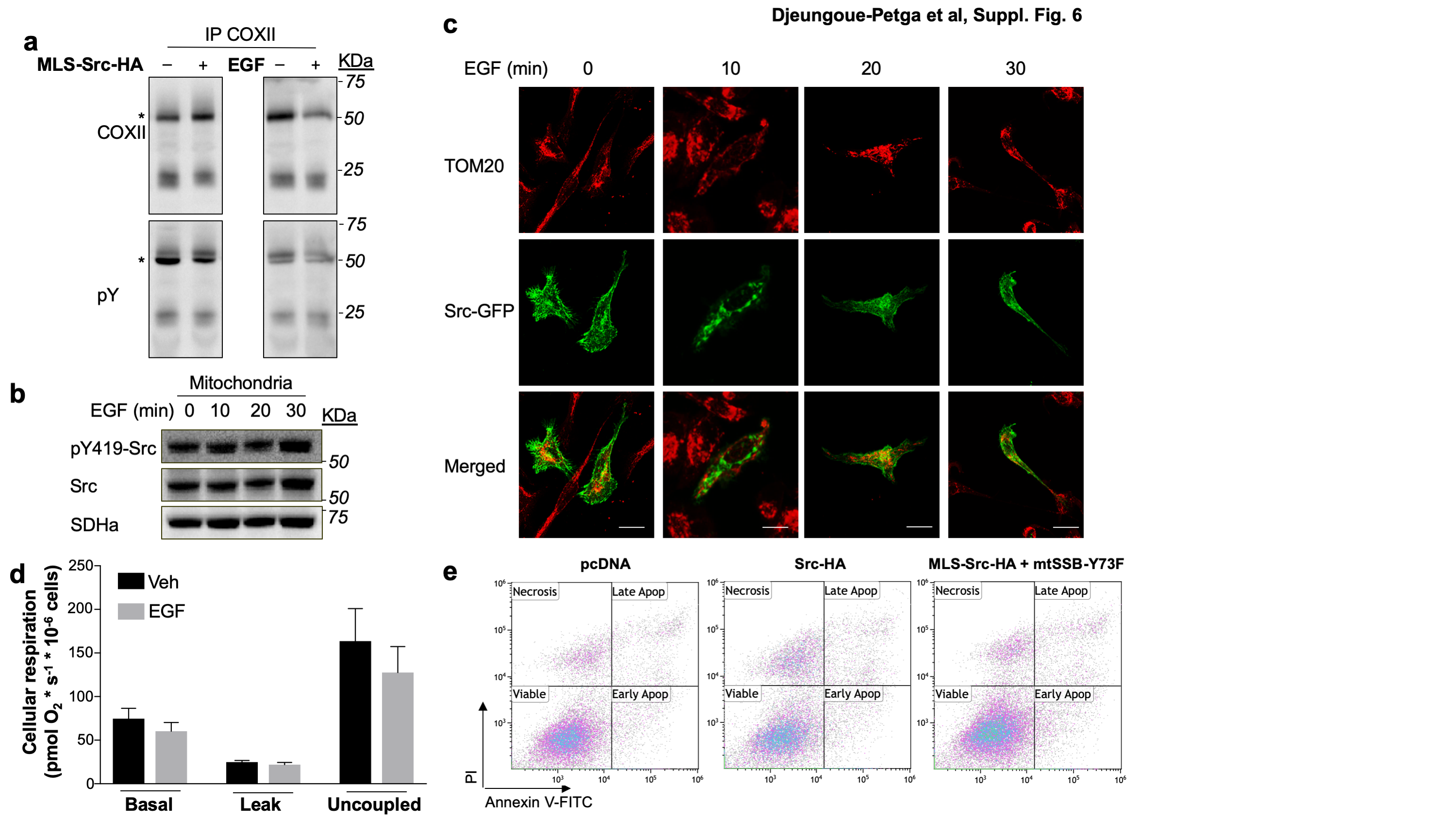

Supplement: Supplementary file 6 — Supplemental Figure 6 [file 41419_2019_2134_MOESM6_ESM.tiff]
