# Supplementary material for: Intramitochondrial Src kinase links mitochondrial dysfunctions and aggressiveness of breast cancer cells
Source: Cell Death Dis. 2019 Dec 9;10(12):940. doi: 10.1038/s41419-019-2134-8 (PMC6901437; doi:10.1038/s41419-019-2134-8)
Supplement: Supplementary file 8 — Author Contribution [file 41419_2019_2134_MOESM8_ESM.pdf]

DECLARATION OF CONTRIBUTIONS TO ARTICLE

ADMC

Manuscript Number:	Journal Name:	
CDDIS-19-1349R	*Cell Death & Differentiation*	(the 'Journal')

Proposed Title of the Contribution:

Intramitochondrial Src kinase links mitochondrial dysfunctions and (the 'Contribution)

Author(s):

MA Djeungoue-Petga, O Lurette, S Jean, G Hamel-Côté, R Martin-Jimenez, M (the 'Authors')

For all *CDD* articles, each person named as an author in the published version must be able to show he or she has contributed substantially to the article.

Authorship credit should be based on 1) substantial contributions to conception and design, acquisition of data, or analysis and interpretation of data; 2) drafting the article or revising it critically for important intellectual content; and 3) final approval of the version to be published. Authors should meet conditions 1, 2 and 3.

Any person who cannot be shown to have made a substantial contribution to the article cannot be listed as an author in the final version. The name of any person who is deemed to have made a minor contribution can, however, appear in the Acknowledgments section of the article.

Please complete the table below to indicate the contributions of all named authors to the manuscript.

Author Full Name:	Specification of Contribution to the Manuscript:
Marie-Ange Djeungoue-	Involved in criteria 1, 2 and 3
Olivier Lurette	Involved in criteria 1, 2 and 3
Stéphanie Jean	Involved in criteria 1, 2 and 3
Genevieve Hamel-Coté	Involved in criteria 1, 2 and 3
Rebeca Martin-Jimenez	Involved in criteria 1, 2 and 3
Marine Bou	Involved in criteria 1, 2 and 3
Astrid Cannich	Involved in criteria 1, 2 and 3
Patrick Roy	Involved in criteria 1, 2 and 3
Etienne Hebert-Chatelain	Involved in criteria 1, 2 and 3

Please complete the table below to indicate the contributions of all named authors to the figures.

Figure 1:
MADJ, OL, MB, SJ, EHC

Figure 2:
MADJ, OL, AC, EHC

Figure 3:
MADJ, OL, SJ, AC, MB, EHC

Figure 4:
MADJ, RMJ, PR, AC, EHC

Figure 5:
MADJ, SJ, AC, EHC

Figure 6:
MADJ, SJ, AC, GHC, RJM, PR, EHC

Signed for and on behalf of the Author(s):	Print Name:	Date:
[signature]	Etienne Hebert Chatelain	10-10-19
